# Javanese *Homo erectus* on the move in SE Asia *circa* 1.8 Ma

**DOI:** 10.1038/s41598-022-23206-9

**Published:** 2022-11-08

**Authors:** Laurent Husson, Tristan Salles, Anne-Elisabeth Lebatard, Swann Zerathe, Régis Braucher, Sofwan Noerwidi, Sonny Aribowo, Claire Mallard, Julien Carcaillet, Danny H. Natawidjaja, Didier Bourlès, ASTER team, Georges Aumaitre, Didier Bourlès, Karim Keddadouche

**Affiliations:** 1 ISTerre, CNRS, IRD, Univ. Grenoble Alpes, 38000 Grenoble, France; 2 School of Geosciences, The University of Sydney, Sydney, NSW 2006 Australia; 3 CEREGE, Aix-Marseille Université CNRS-IRD-Collège de France-INRAE, Technopôle de l’Environnement Arbois-Méditerrannée, 13545 Aix-en-Provence, France; 4 Research Center for Archaeometry, National Research and Innovation Agency (BRIN), Jakarta, Indonesia; 5 Research Center for Geological Disasters, National Research and Innovation Agency (BRIN), Bandung, Indonesia

**Keywords:** Geomorphology, Archaeology, Animal migration

## Abstract

The migration of *Homo erectus* in Southeast Asia during Early Pleistocene is cardinal to our comprehension of the evolution of the genus *Homo*. However, the limited consideration of the rapidly changing physical environment, together with controversial datings of hominin bearing sites, make it challenging to secure the robust timeline needed to unveil the behavior of early humans. Here, we reappraise the first appearance datum of Javanese *H. erectus* by adding the most reliable age constraints based on cosmogenic nuclides $^{10}$Be and $^{26}$Al produced *in situ* to a compilation of earlier estimates. We find that *H. erectus* reached Java and dwelled at Sangiran, Java, *ca.* 1.8 Ma. Using this age as a baseline, we develop a probabilistic approach to reconstruct their dispersal routes, coupling ecological movement simulations to landscape evolution models forced by reconstructed geodynamic and climatic histories. We demonstrate that the hospitable *terra firma* conditions of Sundaland facilitated the prior dispersal of hominins to the edge of Java, where they conversely could not settle until the Javanese archipelago emerged from the sea and connected to Sundaland. The dispersal of *H. erectus* across Sundaland occurred over at least tens to hundreds kyr, a time scale over which changes in their physical environment, whether climatic or physiographic, may have become primary forcings on their behavior. Our comprehensive reconstruction method to unravel the peopling timeline of SE Asia provides a novel framework to evaluate the evolution of early humans.

## Introduction

*When and how did* *Homo erectus* *disperse in SE Asia?* The first part of the question, *when*, has received great attention since the early 20$^{th}$ century discovery of fossil remains in Java^1,2^. The second part, however, has mostly been overlooked even though understanding *how* *H. erectus* dispersed holds clues not only on the climatic^3^, ecological^4^, geological^5^, or physiographic^6,7^ environment suitable for the dispersal of hominins, but also on some of the physical and behavioral characteristics of early humans. This question is all the more relevant given the singular place that Javanese *H. erectus* occupy, not only as a historical landmark^1^, but also owing to their cardinal position at the southeastern end of the realm of Early Pleistocene hominins. Here, we jointly tackle both parts of the question: first, we reappraise the age at which *H. erectus* colonized the region and second, we evaluate their migration pathways using paleo-environmental and ecological modeling techniques.

**Figure 1:**
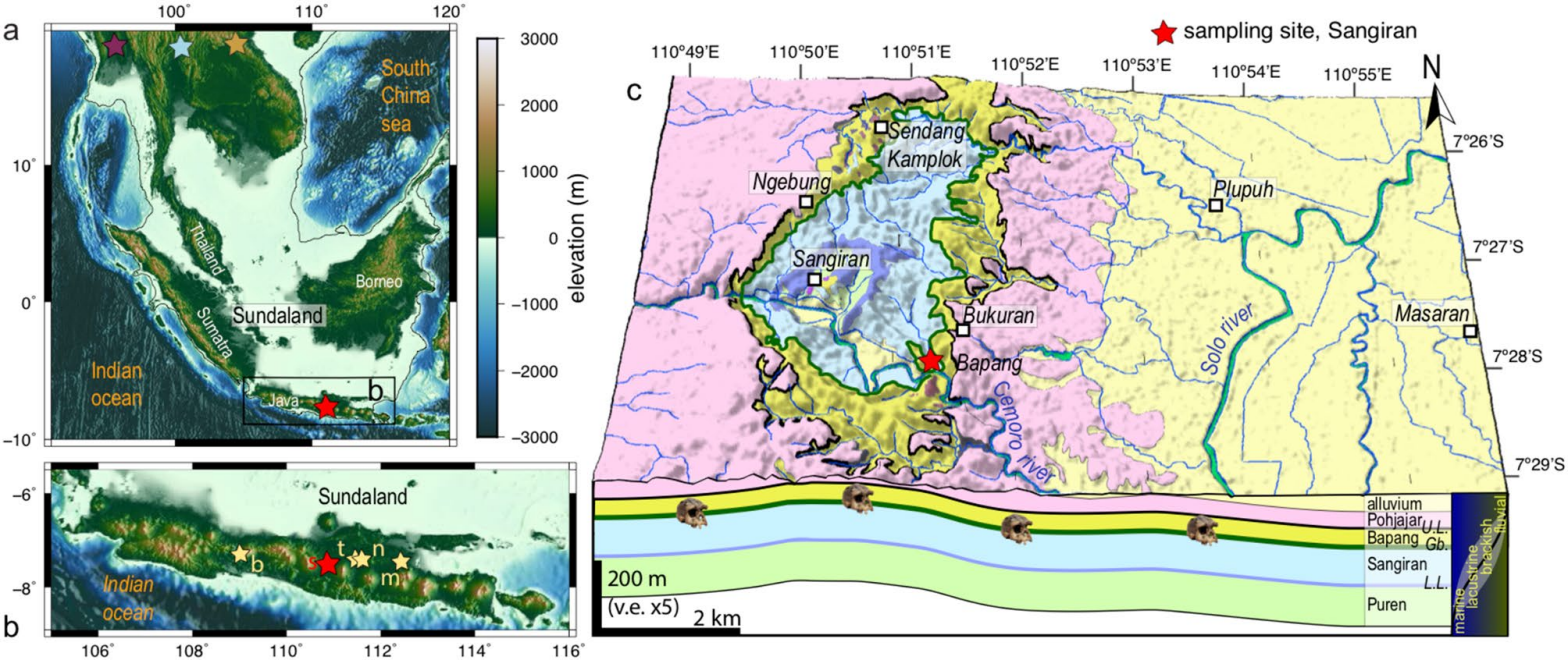
Geology of Sangiran. (**a**) Regional view. Black line delinates continental Sundaland (120 m isodepth). Stars indicate possible northern entry points (thereafter set to Myanmar, Thailand and Vietnam) for *H. erectus*. (**b**) Java island and main biostratigraphic sites (hominin-bearing sites: Sangiran (s), Mojokerto (m), Trinil (t), Ngandong (n); and faunal site Bumiayu (**b**). (**c**) 3D view of the Sangiran dome. Geological map redrawn from^15,22^. Red star locates Bukuran creek outcrop; skull is Sangiran-17^23^. Maps on panels (**a**,**b**) were created using GMT 5 (www.generic-mapping-tools.org)^24^; on panel (**c**). using ArcGIS (www.arcgis.com).

In the very dynamic landscapes of Java and Sundaland (Fig. 1), constantly reshaped by the joint action of geodynamics and climate during the Quaternary^7,8^, a precise knowledge of the chronological framework is requisite to reconstruct the physical environment. We therefore directly dated the earliest arrival of *H. erectus* in Java, which defines the baseline that we then use to assess the past physiography and dispersal routes from mainland Asia to Java. Javanese site Sangiran (Fig. 1) is pivotal in that respect because it yields the largest number of hominin finds, and also because it counts amongst the earliest *H. erectus* bearing sites in SE Asia. Reconstructing the pathways and drivers of hominin dispersal across Eurasia often relies on the purported age of first appearance of *H. erectus* in Sangiran^9,10^, which was commonly bracketed between 1.5 Ma and 1.7 Ma based on $^{40}$Ar/$^{39}$Ar and paleomagnetic dating^11–14^. However, a recent study opposed a younger age of $\sim$1.3 Ma, based on U-Pb radiometric dating and zircon fission tracks^15^.

Dating in Java is notoriously difficult owing to the scarcity of datable material in the volcano-clastic sediments that would unambiguously determine the age of arrival of *H. erectus* with an unbiased method, but also because of the geological setting of the Sangiran dome (Fig. 1c). Despite its tectonic deformation providing good exposure of fossiliferous layers, sedimentary deposits are often inconveniently reworked. Yet, within the available dataset, only the latest dating^15^ is in fact difficult to reconcile with the others. While the large uncertainties and the overdispersion of the data revealed by the mean square weighted deviation (Fig. 3 in^15^) may explain this outlier, their study indisputably revives the controversy. In addition, most previous dating methods suffer from large uncertainties arising from the poor constraints on the pre-burial history. Instead, Terrestrial Cosmogenic Nuclide dating (TCN) is, to our knowledge, the only available method to focus on the burial time and thus to minimize uncertainties associated to the pre-burial history. For that reason, this technique is increasingly applied in archaeology and paleoanthropology, and sheds new lights on hominin dispersal^16–21^. Here, we date the first appearance of Javanese *H. erectus* using TCN relying on the concentration of two *in-situ* produced cosmogenic nuclides ($^{10}$Be and $^{26}$Al) in quartz grains from the sedimentary layers of interest. These isotopes accumulate in the grains when they are exposed to cosmic radiations during exhumation and transport phases, and conversely decay when the grains are shielded during burial periods. The burial duration in terrigenous sediments, and exhumation history until sampling, is thus encoded in the sediments by the amount of each isotope they contain.

A refined knowledge of this time period is crucial to decipher the dispersal and behavior of Javanese *H. erectus* for at least two reasons. First, depending on this age, it is unclear if Javanese *H. erectus* directly arrived along the coastlines in a single *out-of-Africa* episode, or discontinuously stemmed from smaller size groups that expanded earlier in China^25,26^; it implies that early humans could have entered Sundaland from different points in mainland Asia (Fig. 1a). Second, the subsequent trajectories of Javanese *H. erectus* across Sundaland were conditioned by the extremely transient paleoenvironmental conditions. While it is at present-day largely flooded, Sundaland was permanently continental during Early Pleistocene^6^; conversely, while Sundaland was slowly drowning, Java was an uplifting chain of volcanoes emerging from a shallow sea. The settlement of terrestrial faunas and hominins in Java occurs soon after the transition from marine to more terrestrial environments^2,4,11,13,27,28^. Within this broad geographical framework, the direction and pace of movements of *H. erectus* were driven by the contingencies of the local physical environment at the time of their migration, as defined by the river network, relief, and vegetation cover of Sundaland and Java.

To quantitatively unravel their migration pathways across this complex environment, we build upon two recent advances in paleo-environmental and ecological modeling techniques. First, we reconstruct past physiographies over geological time, accounting for the joint effects of geodynamic deformations and climatic forcings, by applying a Landscape Evolution Model (LEM). Concurrently, an array of mechanistic models have emerged from conservationist studies^29–31^ to simulate contemporary species displacements across a given landscape, based on a series of morphometric biases that prompt or hinder the motion of species, like relief and drainage, but also climate or vegetation cover. Here, we reconstruct the past physiography using the LEM *goSPL*^32^ that simulates the joint effects of erosion, sediment transport and deposition on the relief and drainage network with an adequate resolution ($\sim$500 m) to address biogeographical purposes^7^. The modelled landscape is sculpted at the time of hominin migration by the interplay between geodynamics -which deforms the surface of the Sunda shelf^6,7,33^- and climate evolution^34^ -which sets the amount of precipitation. The reconstructed landscape then defines the environmental conditions for the mechanistic model of ecological displacement *SiMRiv*^30^, in which the random trajectories of species are conditioned by landscape complexity, perceptual range, and partial memory of past displacement (semi-correlated Lévy-flight)^30^. Applying this approach to the longterm dispersal of *H. erectus* across Sundaland, we opt for a probabilistic assessment of a large set of simulations (3000 realizations with 5 millions iterations each), which we use to evaluate the travel distances and times, and the likelihood of the presence of hominins at any given location within the region. For reference, we additionally compute the least-cost path end-member solution (upon the incongruous hypothesis that hominins had a destination and a roadmap).

## Results

### Dating Sangiran

**Figure 2:**
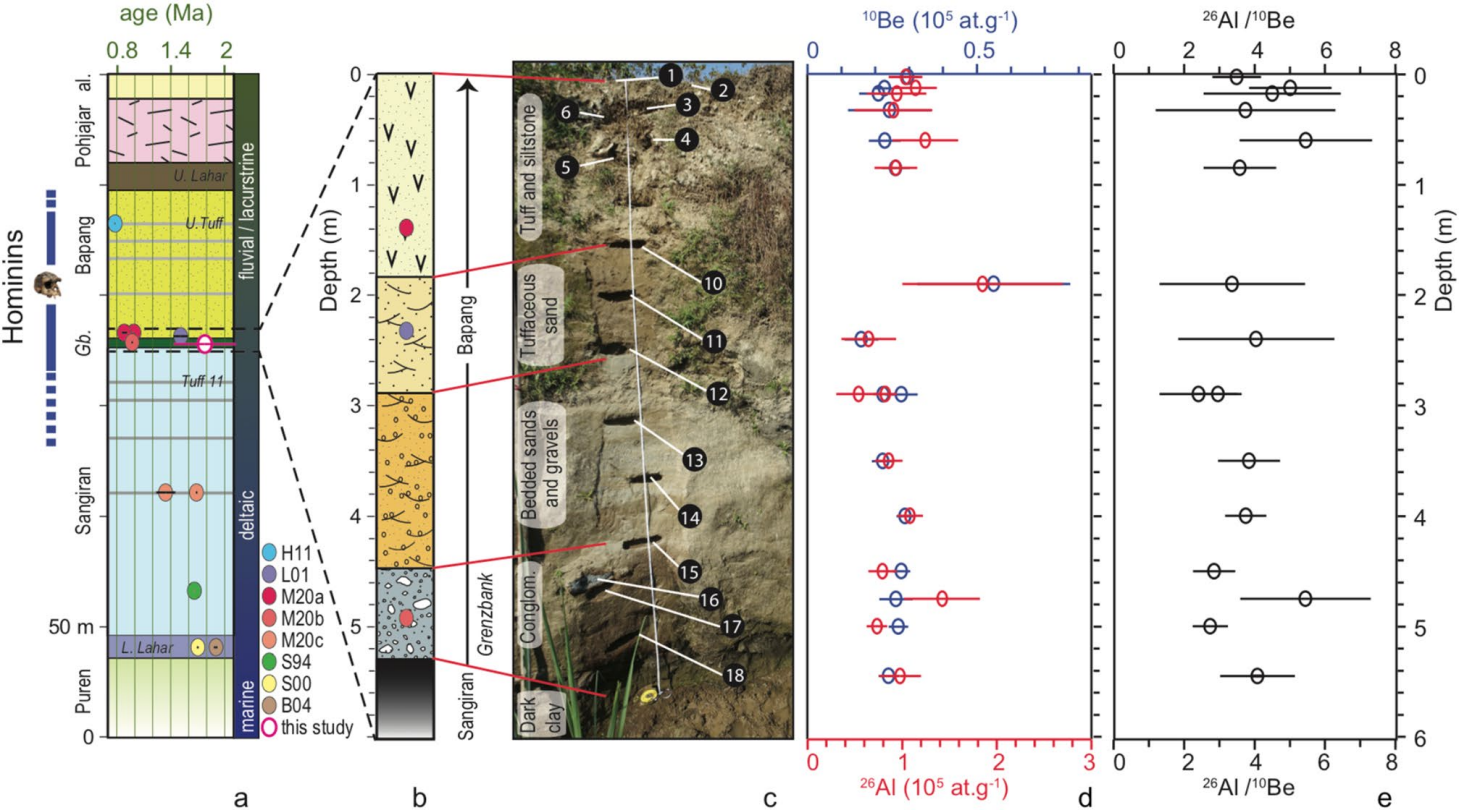
Bukuran sampling site, stratigraphic setting, and analytical results. (**a**) General lithostratigraphy of the Sangiran Dome^15,22,35^, depositional environment, and available datings$\dagger$. Blue bars indicate proven occurrence of *H. erectus* fossils remains, skull is Sangiran-17^23^. (**b**) Stratigraphic log of the sampling site. (**c**) Sampling site, lithologies, and sample location (labeled by their sample name suffix: *1* is SAN18-1). The Bukuran stream flows at the bottom of the outcrop. (**d,e**): $^{10}$Be and $^{26}$Al concentrations and $^{26}$Al/$^{10}$Be ratios over depth. Red and blue dots are $^{26}$Al and $^{10}$Be concentrations (S.I. Table 1). *L. Lahar*: *Lower Lahar*; *U. Tuff*: *Upper Tuff*; *al.*: alluvium; *Gb.*: *Grenzbank* unit. $\dagger$ Earlier datings: *H11*, paleomag^35^. *L01*, $^{40}$Ar/$^{39}$Ar^11^ ; *M20a*, U-Pb and ZFT^15^; *M20b*, U-Pb^15^; *M20c*, U-Pb and ZFT^15^; *S94*, $^{40}$Ar/$^{39}$Ar^12^; *S00*, $^{40}$Ar/$^{39}$Ar, paleomag.^13^; *B04*, $^{40}$Ar/$^{39}$Ar^14^.

We dated the earliest episode of continentalization of the Sangiran Dome (Fig. 2a) in Java island, that was colonized by hominins shortly after^13^. The general lithostratigraphy spans more than 2 Ma^36^. The marine Puren *fm.* forms the earliest layers. It is overlain by the Sangiran *fm.*, wherein the earliest terrestrial faunas are found in the *Lower Lahar* unit^2^. Hominin fossils appear in the uppermost part of the Sangiran *fm.* where shallow marine environments gradually give space to more terrestrial settings. The conglomerates of the *Grenzbank* unit, at the base of the Bapang *fm.* mark the onset of more permanent and hospitable conditions. The overlying Bapang *fm.*, predominantly fluviatile, hosts the vast majority of *H. erectus* finds until the *Upper Tuff* unit. It is unconformably overlain by the Pohjajar *fm.*, and ultimately by recent alluvium from the Solo river; to date, no hominin fossil has been retrieved from these uppermost formations. Following their deposition, the Bapang and Pohjajar formations have been eroded during the deformation of the Sangiran dome, exposing the deeper fossil-bearing formations. In order to most comprehensively evaluate the chronology of the events, we benefit from available ages for the entire stratigraphic series that we complement by TCN dating of the critical *Grenzbank* unit.

Samples were extracted in the Bukuran creek in the SE of the Sangiran dome (Fig. 1c), less than 30 m north of the find spot of a maxilla^27^, at the exact location of a section that was previously dated with discordant ages at $\sim$1.5 Ma^11^ and $\sim$0.9 Ma^15^. The local stratigraphy (Fig. 2b,c) has been documented in great details^15,27^, and corresponds to the base of the Bapang *fm.*, the basal conglomerate being the *Grenzbank* unit, and the dark clay the uppermost unit of Sangiran *fm*.

$^{10}$Be and $^{26}$Al concentrations and ratios do not show significant depth dependence (Fig. 2d,e), while an exponential depth decrease of cosmogenic isotope concentration could instead be expected *a priori* due to the depth attenuation of the incoming cosmic ray particles (Methods). This uniformity results from the long-lasting denudation of the $\sim$100 m thick layers of the Bapang and Pohjajar formations: This episode, which necessarily postdates the deposition of Pohjajar *fm.* at 0.15 to 0.25 Ma^36^, corresponds to mean denudation rates of 400 to 600 m/Ma. As it proceeds, this sustained yet recent exhumation advects and constantly trims the uppermost, $^{10}$Be and $^{26}$Al enriched layers while they accumulate cosmogenic isotopes. Radioactive decay is modulated by post-burial production during the final stage of exhumation, which then has to be accounted for^18,20^. Inverting $^{10}$Be and $^{26}$Al concentrations yields a mean burial age of 1.78±0.20 Ma, pre-burial denudation rates ranging from 21 to 80 m/Ma, and post-deposition denudation rates from 334 to 608 m/Ma (Methods). These mean denudation rates are consistent with geological estimates of erosion rates following the deposition of the Pohjajar *fm.*, and the tenfold increase of pre-burial to post-burial rates is consistent with the respective lithologies of the source rocks (volcanic rocks) and of the unconsolidated Pleistocene sediments. For reference, we also computed the minimal burial age, given by the end-member case considering the (quasi-)instantaneous removal of tens of meters of sediments over the outcrop, while ignoring the longer term erosion of the Bapang and Pohjajar formations. In that case, only the time span since deposition sets the present-day ratios; this hypothesis yields a minimum weighted mean burial duration of 1.45±0.10 Ma (Methods).

This TCN age (1.78±0.35 Ma) of the *Grenzbank* unit arguably belongs to the older end of the range of earlier estimates (Fig. 2a). The dated unit is bracketed by layers of compatible ages, from the base of the Sangiran *fm.* ($^{40}$Ar/$^{39}$Ar and paleomagnetic ages of $\sim$1.9^14^ and $\sim$1.7 Ma^12,13^) and from its core (U-Pb ages of $\sim$1.7 Ma^15^), to the *Upper Tuff* unit in the upper part of the Bapang *fm.* (paleomagnetic ages, $\sim$0.8 Ma^35^). Although the new TCN dating advocates for an older age, it remains compatible, within uncertainty, with the commonly used $\sim$1.5 Ma $^{40}$Ar/$^{39}$Ar age obtained at the same site and stratigraphic position at the base of the Bapang *fm.*^11^. Conversely, this TCN age contradicts recently estimated ages of $\sim$0.9 to 1.0 Ma for the bottom part of the Bapang *fm.* and *Grenzbank* unit (U-Pb and ZFT^15^) and earlier reported age estimates for the Bapang *fm.* of $\sim$0.8 from the northern Sangiran Ngebung site^37^. Besides these outliers, the overall contemporaneity of most ages below the lower Bapang *fm.* can be explained by the rapid sedimentation in the shallow marine to swampy environments of the Sangiran *fm.*, which likely laid the sedimentary pile in less than 100 kyr. These layers thus only represent a snapshot in the history of Javanese *H. erectus*, for which the oldest age provides a conservative estimate of the first appearance datum, *ca.* 1.8 Ma. Conversely during the deposition of the more lacustrine and fluviatile Bapang *fm.*, which may span up to 1 Ma until the last appearance of *H. erectus* in Sangiran *ca.* 0.78 Ma^35^, sedimentation rates are much lower. It implies that, as a simple effect of sedimentation rates, hominin remains could be more diluted in the fast sedimenting Sangiran *fm.* (likely above 1 mm/a) than in the slow-sedimenting Bapang *fm.* (possibly as low as $\sim$0.05 mm/a), consistent with the distribution and frequency of remains in these in the two formations. More generally, the highly variable sedimentation rates of the available archive might bias the interpretation of the taphonomic, paleo-anthropological, paleo-environmental and paleo-ecological conditions at the time of first appearance of *H. erectus*.

### Peopling Sundaland to Java

The slow drowning of the shallow Sunda shelf implies that the entire Sundaland was subaerial during most of the Pleistocene^6^. In order to reconstruct the landscape at the time when *H. erectus* reached Java, we compiled and interpolated estimates of uplift and subsidence rates^7,33^ over the entire region (Methods). The backward integration of these rates over time allows to restore the paleo-elevation of Sundaland at large spatial scales. In addition, in order to comprehensively retrodict the physiography at the local scale and determine the migratory behavior of *H. erectus*, we model the surface processes overprint on this long-wavelength relief using LEM *goSPL*^32^. We performed these reconstructions opting for a baseline age of 1.8 Ma, which, based on the TCN age and existing earlier dating, is most representative of the period of dispersal, but uncertainties in the ages and vertical land motions of course propagate into our physiographic reconstructions.

**Figure 3:**
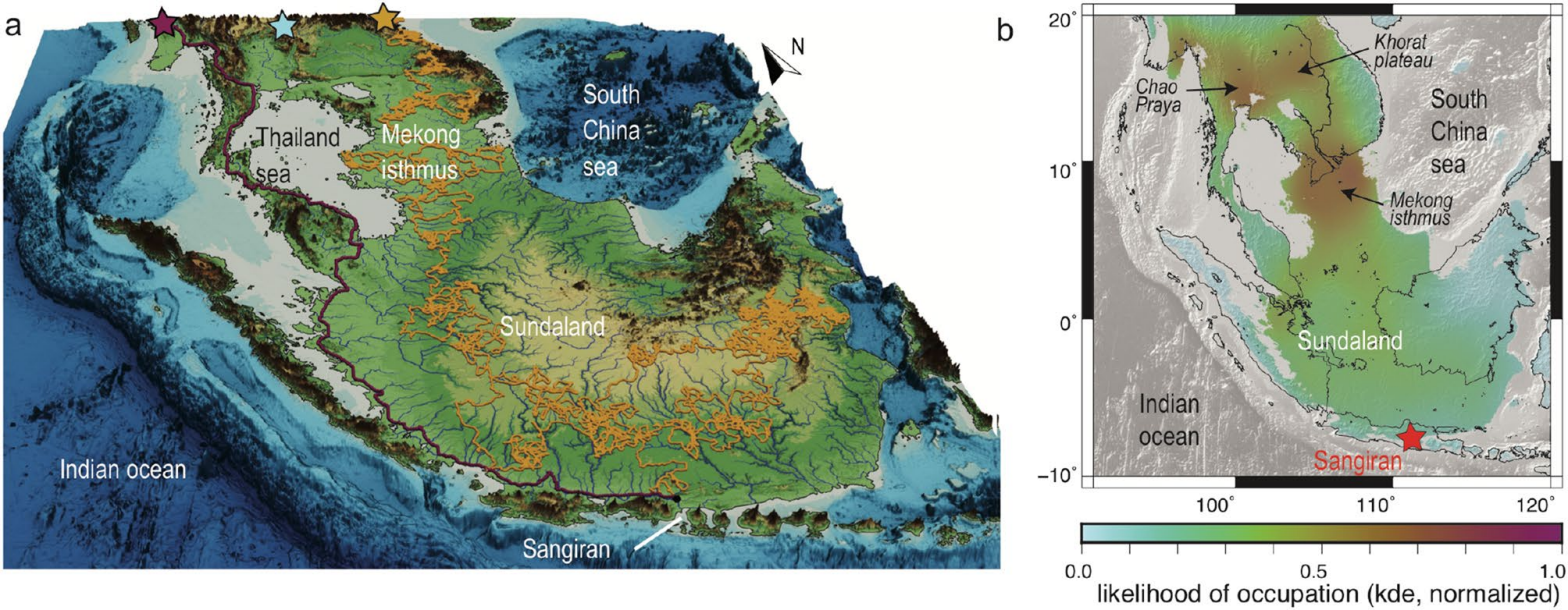
Migration pathways of *H. erectus* across Sundaland during Early Pleistocene. (**a**) Reconstructed landscape *ca.* 1.8 Ma (model *goSPL*^32^). The underlying physiography, with overprinted modeled rivers, converts into a map of resistance to displacement that serves to compute the migration pathways, either using a least-cost path algorithm shown for the Myanmar entry point (purple), or a mechanistic movement model (*SiMRiv*^30^) exemplified for the Vietnam entry point (orange). Blue star locates the Thailand entry point. (**b**) Statistical analysis of the pathways of *H. erectus* migration to Sangiran, from 3000 simulations (1000 per entry point). The kernel density estimation (kde, normalized) indicates the likelihood that hominins came across a given region (combined for the three entry points). Map on panel (**a**,**b**). were respectively made using Paraview (www.paraview.org) and GMT 5 (www.generic-mapping-tools.org)^24^.

These reconstructions primarily show that Sundaland was permanently continental *ca.* 1.8 Ma (Fig. 3a), regardless of glacio-eustatic sea level oscillations, while tectonic uplift in the Indonesian arc completed the land bridge from mainland Asia to the emerging relief of the Javanese archipelago. The elevation gently decreased from more than 1000 m in the East towards the lowlands of what can be referred to as the “Mekong isthmus” that separated the internally drained Thailand sea (now Gulf of Thailand) from the South China Sea to the North, and towards the marginal seas and alluvial plains that border the inner margins of Sumatra and Java to the Southwest. The drainage network finely dissected the surface of the continent, with a set of major rivers, radially diverging from the core of Sundaland outwards, that incised the shelf; in the South in particular, N-S trending rivers profoundly imprinted the landscape (Fig. 3a and S.I. Fig. 1B). Because of their sizes (the largest computed discharge compares to that of the present-day Yangtze), these rivers might have acted as barrier to migration and could have channelized terrestrial faunas and early humans^38^ towards the shallow marginal seas that separated the shelf from the emerging landmasses of Java, where marine conditions prevailed until Early Pleistocene (Fig. 3). The protracted subsidence of Sundaland, stimulated by the slow deformation of the Earth interior^33^ changed the landscape during mid-Pleistocene^7^. Lowlands gradually invaded most of the shelf, with only a limited set of rivers draining towards the South China, Flores, and Andaman seas, followed by ever more pervasive marine incursions over the latest interglacial stages.

Once the land bridges connecting Java to South Sundaland were opened during Early Pleistocene, *H. erectus* reached Java, neither as seafarers nor islanders, but as continental walkers. The stratigraphic record in Java ubiquitously indicates that terrestrial faunas and *H. erectus* dwelled as soon as the environment became continental enough to support them^13^. Emersion was triggered by Plio-Pleistocene tectonic shortening and uplift. It started in East-Central Java -near Sangiran- and gradually propagated westward^39,40^ and northward, suggesting that the Sangiran dome emerged and hosted hominins and terrestrial faunas earlier than the northern and western counterparts, for instance near the more recently occupied site of Trinil^41,42^ (Fig. 1b).

In order to develop a quantitative approach of the peopling of Sundaland and Java, we converted this contrasted physiography into a cost map of landscape complexity, that quantifies the resistance of the physiography to the displacement of species, integrating the effects of slope, distance to water bodies (rivers and shores), and river size (Methods). Both least-cost paths and random (Markovian) walks eventually lead hominins from mainland Asia to Java, but with extremely variable routes and travelled distances (Fig. 3a). Depending on the entry point (Myanmar, Thailand or Vietnam), least-cost paths show two preferential routes, either following the western coastline of the epicontinental Thailand sea or opting for an eastern path through the “Mekong isthmus”.

Markovian walks instead are more exploratory (see animated examples in Supplementary Information), and show that the entire region was made available to hominins, albeit with a variable probability. The likelihood that the species came across a given location (revealed by the kernel density estimation of all 3000 modeled random walks, Fig. 3b) unsurprisingly reaches the highest values in the northern part of the region, near their entry points. En route to Sundaland, while the leasts cost paths includes a western trajectory through the Malay peninsula, the probabilistic approach instead indicates that this western option is unlikely due to the rugged topography of the Malay peninsula. Instead, the “Mekong isthmus” channelized them between the Thailand and South China seas. In these lowlands, landscape biases are low (absence of relief, favorable drainage network, see Methods), which further reinforces the likelihood of hominin occurence. Likewise, the likelihood is slightly higher in the lowlands of eastern Sumatra than in more mountainous regions of Sundaland, and in the North, in the flat Chao Praya basin and Khorat plateau of Thailand (Fig. 3b). While these results indicate that peopling Sundaland was ubiquitously possible, the likelihood that hominins joined the rims of Sundaland is however relatively low. This result, seemingly at odds with the Javanese yield of fossil remains, could reveal that our reconstructions and parametrizations are inadequate to the point of being misleading, and that better accounting for habitat suitability, using species distribution modeling in particular, would yield alternative and more compatible solutions. Alternatively, this incongruence can be simply explained by the inconvenient concealment of fossil bearing sedimentary layers in subsiding, fast sedimenting basins (as in Thailand) or below sea level (as in the “Mekong isthmus”).

**Figure 4:**
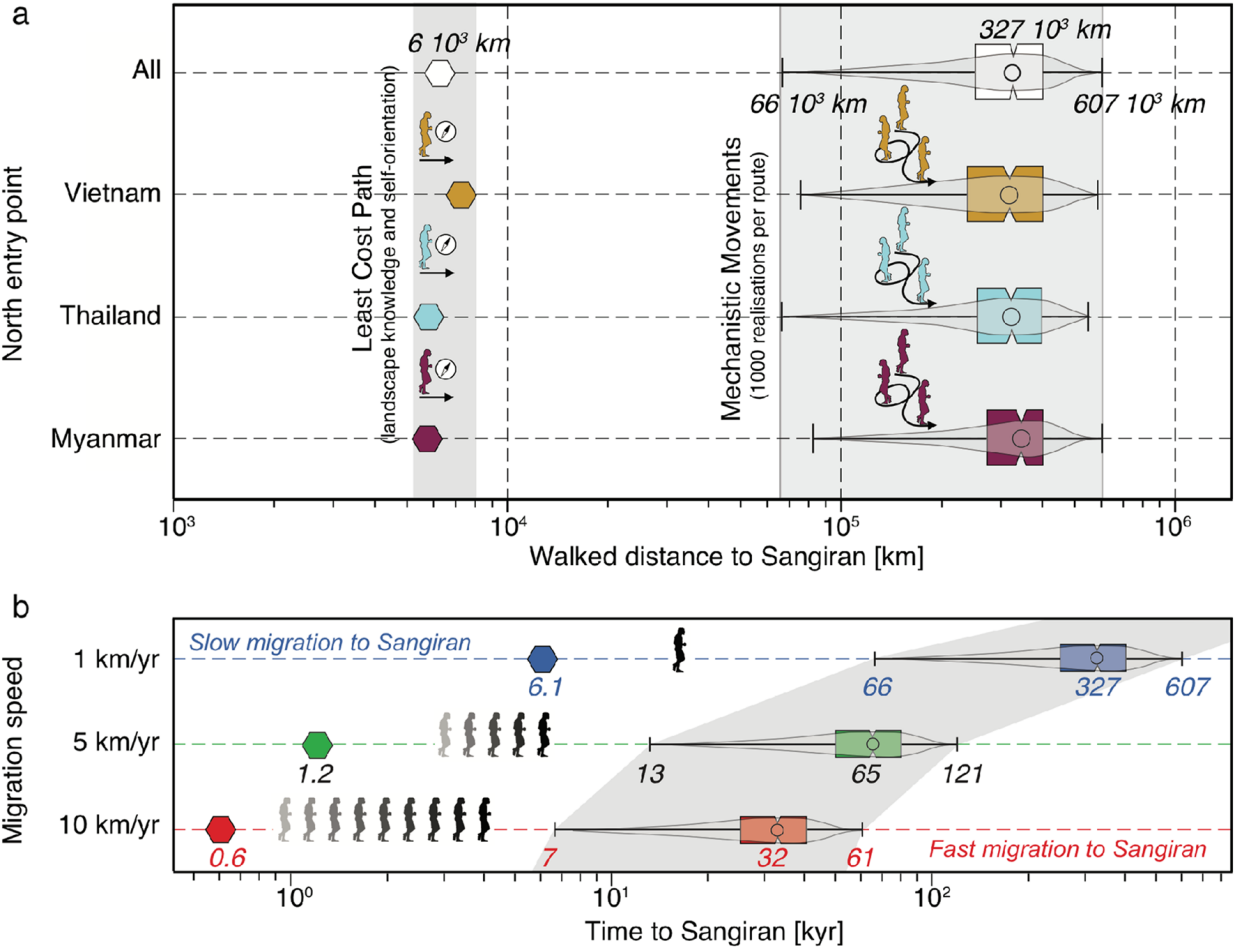
Travelled distance and time of *H. erectus* migration to Sangiran, statistical analysis (3000 simulations, 1000 per entry point - Myanmar, Thailand, and Vietnam). (**a**) Migration distance from the least-cost path (LCP) analysis and successful mechanistic movement realizations (Markovian random walk) for the three entry points, and all combined. (**b**) Travel times, for three dispersal speeds (slow: 1 km/yr^43^, medium: 5 km/year^44^, and fast: 10 km/yr^45^).

How long did the journey take? While least-cost paths are $\sim$6,000 km long on average from mainland Asia to Java (Fig. 4a), the mean travelled distances are $\sim$50 times longer for Markovian walks (327$\times 10^3$ km), regardless of the north entry point, and almost always in excess of 100$\times 10^3$ km. The corresponding time span depends on the migration speed. Estimates are available for more recent palaeolithic hominins, ranging from 1 to 10 km/yr^43–45^. At similar speeds, the least-cost paths convert into an optimal travel time of 0.6 to 6 kyr, which is thus the minimal time to cross Sundaland. Markovian walks imply much longer durations before hominins reached Java, between 25-40 kyr at fast moving rates, and 250-400 kyr at slow moving rates (Fig. 4b). These relatively long delays at average or slow moving rates are difficult to reconcile with the early arrival to Java *ca.* 1.8 Ma, following departure from Georgia or China only a few 100 kyr before. We suggest that because these characteristic travel times scale with the characteristic time scale of changes of the physical environment, external forcings impelled hominins to migrate and boosted their displacement. Climatic variations notoriously impact the habitats of hominins^46,47^, but here we suggest that the fast physiographic changes of geodynamic origin and environmental stochasticity could have also prompted the dispersal of *H. erectus*.

## Discussion

Eugène Dubois^1^ wondered early on whether his revolutionary findings in Java, as opposed to his unfruitful attempts in Sumatra, were due to the habitats of early humans or to their preservation and outcrop. This question is in fact a general one, on which our joint analysis of the chronology, physical environment, and dispersal trajectories brings a new light on: Hominin finds in Java mark the onset of continental conditions there *ca.* 1.8 Ma rather than the timing of their migration across SE Asia. The high probability that earlier groups of hominins thrived in the nearby Sundaland and were ready to disperse in Java, only waiting for more continental conditions in the Javanese archipelago, suggests that the immediate ancestors of Javanese *H. erectus* could be abundantly buried in the sediments of the nearby Sunda shelf more than exposed in the Indonesian arc. During Early Pleistocene, *H. erectus* likely clustered in lowlands during the peopling of Sundaland, in particular in the “Mekong isthmus” that connected mainland Asia to the Sunda shelf, or in a few hotspots in mainland Asia. These sites are currently buried underneath sediments or seawater, or both, which explains that fossil finds are few elsewhere than Java.

This analysis is based on a large number of set of random walks across the region. While it advantageously indicates the probability distribution of the trajectories, distances and tempos, a corollary limitation arises from the unaccounted possibility that the peopling of SE Asia could have been the case of a limited number of groups only. It would imply that a limited number of stochastic events, including the random selection of low cost paths, could have been influential on the final peopling of the region.

While the impact of physiography on hominin evolution has been identified^48^, this case study of the iconic Javanese *H. erectus* emphasizes the need to tie a quantified reconstruction of the past physiography to a robust time frame before attempting to unravel the migration trajectories. This novel approach sheds new lights on the dispersal of the genus *Homo*, by recasting hominins within their regional environmental framework. First, our results emphasize the role of the physiography -lowlands, rivers, and reliefs in particular- in controlling hominin habitats. Second, and perhaps more importantly, they highlight the impact of the transience of the environmental factors, by demonstrating that hominin dispersal operates at the same characteristic time scale than that of physiographic changes, which could have spurred their displacement.

These results open new perspectives to untangle the physical and behavioral capacities of *H. erectus* within their changing physical environments. We find that *H. erectus* migrated across Sundaland at remarkably fast rates (>10 km/yr) with respect to their prior journey across Eurasia, and to the displacement rates of other lineages of the genus *Homo*. We suggest that the quickly changing physiography of Sundaland could have prompted their fast migration, which implies that extrinsic (environmental) forcings prevailed over intrinsic factors (either cultural or demographic)^10^. This proposition invites to go beyond the current study and comprehensively investigate the joint evolution of the entire ecological and physical -climatic and physiographic- environments. Physiographic changes in SE Asia triggered a chain of modifications that impacted the behavior of *H. erectus*, including the regional climate and its feedback relationships with the vegetation cover^49^, and ultimately on the foodchain on which hunter-gatherers relied^50^. In order to more thoroughly address the impact of this changes on habitat suitability, species distribution modeling would be a natural avenue to more precisely reconstruct the evolution of early humans^47,51^.

At a more general scale, our TCN ages obtained for the first appearance datum of *H. erectus* in Sangiran advocate for an early peopling of Sundaland by *H. erectus*, contemporary with their Chinese^26,52^ and Georgian^53,54^ counterparts, whose endocranial capacity was much smaller^54^ than specimens from Sangiran^55^. These results reignite the long-standing controversy over the origin and dispersal pathways of archaic humans^27,52,56^ and invites a reexamination of the out-of-Africa paradigm, which provides a global roadmap for the dispersal of the genus *Homo*, but which one-way direction may be questioned^12,57–60^.

## Methods

### Terrestrial Cosmogenic Nuclide dating (TCN)

The Bukuran creek outcrop (Fig. 1, red star, $110^\circ 51'18'' E, 7^\circ 27'58.3'' S, 99 m.a.s.l.$) was sampled in July 2018. The 6 m high vertical section (Fig. 2) is continuously rejuvenated by lateral erosion by the Bukuran stream, thus offering ideal conditions for TCN application. $^{26}$Al/$^{10}$Be burial dating^16,18–20^ is based on the relative decay of $^{26}$Al and $^{10}$Be cosmogenic nuclides produced *in situ* in quartz (SiO$_2$) minerals upon exposure to cosmic rays. Given the half-lives of $^{10}$Be (1.387±0.012 Ma^61,62^) and $^{26}$Al (0.705±0.024 Ma^63,64^), it is applicable over a time frame of 200 ka to $\sim$6 Ma^16^. During burial, the attenuation of secondary cosmic ray particles with depth is marked by an exponential depth decay of cosmogenic isotope concentration, while posterior erosion flattens out the depth dependence.

The top of the sequence, down to $\sim$1.5 m depth, is composed of a yellowish tuff and quartz poor siltstone. A light pink and yellowish tuffaceous sand, also quartz poor, follows down to 3 m below. Between 3 m and 4.6 m, a bedded sand and gravel layer yields a higher concentration of quartz grains. From 4.6 m to 5.6 m, an indurated conglomerate with a higher concentration of quartz is observed, followed by a layer of dark clays, down to the stream bed. 18 samples have been collected at 15 different depths from the surface (SAN18-1) down to the stream bed (SAN18-18), the deepest samples (SAN-15 to SAN-18) being precisely extracted from the *Grenzbank* unit, as identified in earlier studies^15,27^.

Measured $^{10}$Be and $^{26}$Al concentrations and ratios are depth independent and in agreement (see raw and statistical values in S.I. Table 1). This is explained by low and uniform inherited concentrations, and by long-lasting denudation at a fast enough rate to dampen the depth dependent exposure of quartz grains to cosmic rays. By modeling radioactive decay, we estimate the burial age, pre-burial and post-burial denudation rates^18,65^. We assume that all samples were buried deep enough to be fully shielded from cosmic rays, but account for post-burial production of nuclides since deposition (burial *with* post-production). We also model pre-burial $^{26}$Al/$^{10}$Be ratios (assuming that samples were at steady state before deposition). As all samples underwent the same burial-exhumation history, we inverted for a common burial time and post-production for all samples at once, while leaving individual inheritance independent for each sample. This yields a burial age of 1.78±0.20 Ma ( S.I. Table 1). Denudation rates of the source rocks range from 21 to 80 m/Ma, while post-deposition denudation rates range from 334 to 608 m/Ma with a best fit value of 436 m/Ma. Within the topmost meter of the sequence, the fraction of post-burial $^{10}$Be and $^{26}$Al respectively ranges from 14 to 28% and from 26 to 47%, but then decreases at greater depths to less than 10% for $^{10}$Be and less than 20%, mostly, for $^{26}$Al. Given the inferred high denudation rates, the corresponding time span is on the order of 4000 years.

For reference, we also consider the end-member scenario, wherein the upper part of the profile has been either quasi-instantaneously truncated by several tens of meters (burial *without* post-production), in order to determine the minimal age. Individual minimum burial durations (*i.e.* treating each sample independently) range from 0.58±0.20 Ma to 2.25±1.04 Ma, and pre-burial denudation rates range from 23 to 101 m/Ma (S.I. Table 2). $\chi ^2$ test isolated three outliers (SAN18-2, SAN18-4 and SAN18-16). The remaining 13 samples yield a minimum weighted mean burial duration of 1.45±0.10 Ma.

Chemical sample preparation and $^{10}$Be and $^{26}$Al measurements were carried out at the French Accelerator Mass Spectrometry national facility, ASTER (CEREGE, Aix-en-Provence) following the standard procedure^19^. A challenging aspect for TCN application at the Bukuran creek site is the low quartz content of the fluvio-volcanic sediments. As the amount of quartz mineral is notoriously poor, a mass of 0.5 to 2 kg of sediment per sample was collected, depending on the lithology (S.I. Table 3). Acid attacks were repeated until the maximum amount of pure SiO$_2$ was retrieved, within the availability limit of materials. All samples (besides SAN18-7 to SAN18-9) yielded enough quartz to perform suitable measurements. After purification, the quartz content was dissolved in HF 48% after addition of 150 $\mu l$ of a 3025±9 ppm $^9$Be solution. Natural Al content was determined by ICP-OES using an ICAP6500 from Thermo. BeO and Al$_2$O$_3$ were respectively mixed with niobium and silver powders prior to measurements. Beryllium data were directly calibrated against the STD11 standard^66^ with a $^{10}$Be/$^9$Be ratio of (1.191±0.013) x 10$^{-11}$. Aluminum measurements were performed against in-house standard SM-Al-11, with a $^{26}$Al/$^{27}$Al ratio of (7.401±0.064) x 10$^{-12}$, previously cross-calibrated against the primary standards certified by a round-robin exercise^67,68^. The successive measurement batches are characterized by stable, high level $^9$Be and $^{27}$Al currents during AMS measurements (S.I. Table 3) that attest for their high reliability (despite low $^{10}$Be and $^{26}$Al counts at the AMS receptors). Analytical uncertainties (reported as 1$\sigma$) include uncertainties associated with AMS counting statistics, AMS external error (0.5% for $^{10}$Be), chemical blank measurement, and $^{26}$Al, $^{27}$Al measurements. Measurements of chemically processed blank yield ratios on the order of (2.0±0.75) x 10$^{-15}$ for $^{10}$Be and (2.0±2.0) x 10$^{-15}$ for $^{26}$Al. A sea level high latitude spallation production rate of 4.02±0.32 $at.g^{-1}.a^{-1}$^69^ was used and scaled using Stone’s polynomials^70^. The $^{26}$Al/$^{10}$Be production ratio induced by the standardization used at ASTER is 6.61±0.50.

### Sundaland physical landscape reconstruction *ca.* 1.8 Ma

In order to reconstruct the landscape at 1.8 Ma, we first compiled rates of vertical land motions inferred from geomorphological indicators, stratigraphic, and seismic data (S.I. Fig. 1a adapted from^6,7,33^). This dataset provides discrete informations that we interpolated in order to obtain a continuous map of uplift rates over the region, which backward integration in time provides a paleo-elevation map. To reconstruct a realistic landscape at 1.8 Ma, we ran the Landscape Evolution Model (LEM) *goSPL*^32^ over a 10,000 years period starting from an adjusted paleo-elevation surface (1 km resolution) derived from the uplift map and past absolute sea level, set to -30 m during Early Pleistocene sea level highstands^71^. Continuity of mass is the main equation of the model, and is expressed by:

1 $$\begin{aligned} \frac{\partial z}{\partial t} = U + \kappa \nabla ^2 z + \epsilon P^d (PA)^m \nabla z^n \end{aligned}$$

where *z* is surface elevation (m), *t* is time (yr), *U* is vertical land motion ($\text {m}/\text {yr}$) and $\kappa$ the diffusion coefficient for soil creep (set to $5\times 10^{-3}$ m$^2$/yr). The last term in the right-hand side of Eq. (1) represents fluvial processes based on a stream power law with erodibility $\epsilon$ (set to $4\times 10^{-6}$ m$^{-1}$yr$^{-1}$), dimensionless constants *m* and *n* (empirically set to 0.5 and 1) and water flux *PA* combining upstream watershed area *A* and precipitation *P*. The stream power law here incorporates the effect of local mean annual precipitation rate (obtained from paleoclimate model *HadCM3BL-M2.1aD*^34^), *d* being a positive exponent (set to 0.42), fingerprinted in the paleo-landscape and paleo-drainage maps (see resulting landscape after 10 kyr of concurrent action of riverine and hillslope processes in S.I. Fig. 1b). Both processes erode, transport and deposit sediments with maximum erosion (>500 m) along the Barisan Mountains in Sumatra and deposition (up to 400 m) in several endhoreic lakes and basins across Borneo and East Malay Peninsula (S.I. Fig. 2a).

### Resistance to species displacement from regional physiography

To compute the dispersal of *H. erectus* in Sundaland and its migration to Sangiran, we extracted from the landscape evolution model three physiographic features known to influence early human dispersal^72–75^. These features (S.I. Fig. 2) are then converted into cost values at a $\sim$1 km$^2$ resolution, with high costs corresponding to resistant regions impeding movement. First, we consider endorheic lakes and oceans as absolute dispersal barriers (normalized cost set to 1.0) and assume that major rivers (> 5.5$\times$10$^3$ m$^3$/s - approximately the Niger river flow rates) represent strong barriers to crossing^72^. From our simulation, the largest river flow rate reaches 30.5$\times$10$^3$ m$^3$/s, one sixth of the current discharge of the Amazon river, comparable to the discharge of the Yangtze river. As opposed to earlier studies^72^, we do not deem these major rivers as fully impermeable in the cost calculation, and let their normalized values to vary linearly with flow rates from 0.7 to 0.95. Second, we estimate a riparian buffer zone by computing the distance to rivers over the entire region. In addition to rivers, we also set maritime and lacustrine coastlines as preferential pathways^38^ and add these distances in our cost calculation. Computed distances are then converted into an exponential series^75^, to account for the exponentially increasing difficulty of travel as *H. erectus* moves away from freshwaters^72^. Finally, we account for topographic complexity by calculating the local slope Slopes below 0.5$^{\circ }$ are considered costless, and the normalized cost increases linearly between 0.1 and 0.6 from 0.5$^{\circ }$ to 5$^{\circ }$ and set to 0.8 above 5$^{\circ }$. Combining individual cost from each variable, we obtain a final normalized cost map (S.I. Fig. 1c) that favour routes along riparian areas and coastal plains and avoid hilly and mountainous regions. Assuming foraging time and energy expenditure minimization, this cost map is then used to compute the dispersal of *H. erectus* across South East Asia and colonization of Sundaland.

We then defined three entry points, corresponding to western, central and eastern routes, at places with low resistance to displacement (riparian areas with low topographic complexity). First, from its migration out of Africa via the Levantine corridor, traces of a western route can be found in Riwat, Pakistan^76^, which could lead to the Himalayan foreland, Ganges-Brahmaputra rivers, down to the North of the Irrawaddy delta in Myanmar. Second, hominin fossil remains in south China near Yuanmou^77^, that we found contemporaneous to *H. erectus* from Sangiran, set an eastern route, hypothesizing that the Mekong and Red rivers acted as barriers and channelized early humans to the coast south of the Red river in Vietnam. Last, an intermediate northern route upstream of the Chao Phraya plain in Thailand, hypothesizes that *H. erectus* either crossed the Shan plateau in Myanmar or the Yunnan-Guizhou plateau, bounded by the Salween and Mekong rivers on its western and eastern sides.

### Assuming *H. erectus* omniscience: least-cost paths

A convenient theoretical approach to predict movement of species through a landscape relies on a least-cost path (LCP) analysis^75,78^. Here, LCP are calculated using the Dijkstra’s algorithm accounting for diagonal connectivity between cells and implemented using the scikit-image library^79^. LCPs not only require the positions of the entry points but also the location of the ending point (here set to Sangiran). Both the traveled distance and landscape resistance (S.I. Fig. 1c) determine the most parsimonious route connecting these endpoints. While stochasticity imposes to consider this route as a possible option, LCP otherwise implicitly rests on the unrealistic assumption that the species had a destination, a compass and a roadmap. LCPs thus give a reference for the minimum travelled distance across Sundaland. Estimated walked distances for all 3 cases are comparable (5545, 5620, and 7050 km, respectively for the Myanmar, Thailand, and Vietnam entry points). The western and central routes merge after $\sim$1500 km downstream of their entry points (S.I. Fig. 3), while the eastern route merges with them South of the endorheic Thailand sea, between the Malay peninsula and the “Mekong isthmus” that connected South Vietnam to the Sunda shelf. As expected from the cost surface, LCP follows preferentially alluvial plains and coastal regions. LCP occasionally significant detours from the shortest distance path, specifically where large rivers hamper dispersal and force migration across upstream smaller tributaries of lower discharge.

### Mechanistic movements conditioned by landscape heterogeneity: Markovian walks

Migration models assuming a destination (*e.g.*, LCP) would rarely reflect the actual movements of species across a heterogeneous landscape, which impairs their predictive capacity. In reality, most species progress without a planned destination during their lifespan^80^ and have no memory of longterm prior displacements: their movements are only determined from local information^81^ and immediate prior displacement. To lift the aforementioned LCP limitations, we adapted the recently developed mechanistic model SiMRiv^30^ that simulates spatially explicit stochastic movements (multi-state Markov model^82^) accounting for landscape heterogeneity and perceptual range (*i.e.*, the radius up to which an individual perceives its surroundings) (S.I. Fig. 4a). Here again, the normalized resistance map (S.I. Fig 1c) defines the underlying environmental complexity of the region and *H. erectus* movement is simulated with a two-state movement (Lévy-like walker^83^) that alternates between random and correlated random walks^84^, where the incremental azimuthal direction is correlated with the prior direction (turning angle concentration parameter of the wrapped normal distribution is set to 0.99 in our simulations) (S.I. Fig. 4b). To inform the mechanistic model on the probabilities of changing between the two states, SiMRiv provides a transition matrix (which values are here set to 0.01 and 0.002). We also set the step length (unit of movement) for both states to 100 m in order to avoid unrealistic jumps over high cost cells. Finally, we set the perceptual range to 5 km for both states: the movement decisions at each step accounts for the resistance that the walker *sees* in a 5 km radius around its current location (*i.e.*, it attempts to bypass high-resistance areas within this range, see S.I. Fig. 4b, which illustrates the two-state mechanistic movement for a given realization with multiple phases of acceleration and deceleration as *H. erectus* moves over the heterogeneous landscape). To have statistically significant results, we run 1000 realizations over 5 million steps each for the three routes (Myanmar, Thailand, and Vietnam entry points). One third of the 3000 realizations reaches Sangiran (respectively 29%, 34% and 38%). As expected, Markovian walks to Sangiran are much longer than LCP (LCP lengths amount to 7-9% of the shortest mechanistic realizations - Myanmar: 83,000 km, Thailand: 66,000 km, and Vietnam: 76,000 km - S.I. Fig. 5a). We then compute the likelihood of *H. erectus* occupation at any given location, when combining all realizations successfully passing through Sangiran. We use a kernel density estimate analysis (KDE, S.I. Fig. 5b) weighted by the inverse normalized cost surface, which not only allows to illuminate the most favourable physiographic settings (*e.g.*, riparian areas, lowlands and coastal plains) but also to account for the slower progression (and proportional number of steps) in complex landscapes (S.I. Fig. 4b).

### *H. erectus* migration speed range

Estimating the dispersal velocity across Sundaland remains challenging for *H. erectus*, and we rely on available estimates for better documented, more recent hunter-gatherers hominins. In light of the limited available data on their diet and anatomy, *H. erectus* are also commonly thought to be hunter-gatherers^85,86^, although controversy persists^87^. Recolonization speed estimates for late glacial hunter-gatherers of northern Europe, deduced from reproduction and mobility parameters, occurred at rates of 0.7 to 1.4 km/yr^43^. The distribution of the Clovis-age occupations across North America suggests a speed of 5-8 km/yr^44^. The first peopling of the Americas, and the Neolithic transition in Europe, occurred at rates between 6 and 10 km/yr, based on archaeological boundary conditions^45^. mtDNA variation in isolated populations in southeast Asia shows a migration speed of 4 km/yr for the dispersal of modern humans from Eurasia to Australasia^88^. Based on these estimates, we conservatively assume plausible migration speeds ranging from 1 to 10 km/yr for Southeast Asian *H. erectus*, and use this range to compute the travel time for each of the routes obtained from both LCP and Markovian walks.

## Supplementary Information

Supplementary Information 1.

Supplementary Legends.

Supplementary Video 1.

Supplementary Video 2.

Supplementary Video 3.

## Data Availability

All necessary data are available in the text and tables.

## Code availability

goSPL^32^, is available from https://github.com/Geodels/goSPL and the software documentation can be found at https://goSPL.readthedocs.io. SiMRiv^30^ is available at https://github.com/miguel-porto/SiMRiv.
