# Supplementary material for: Javanese Homo erectus on the move in SE Asia circa 1.8 Ma
Source: Sci Rep. 2022 Nov 8;12:19012. doi: 10.1038/s41598-022-23206-9 (PMC9643487; doi:10.1038/s41598-022-23206-9)
Supplement: Supplementary file 1 — Supplementary Information 1. [file 41598_2022_23206_MOESM1_ESM.pdf]

Supplementary Information : Javanese *Homo* erectus on the move in SE Asia *ca.* 1.8 Ma

Laurent Husson[1*], Tristan Salles[2], Anne-Elisabeth Lebatard[3], Swann Zerathe[1], Régis Braucher[3], Sofwan Noerwidi[4], Sonny Aribowo[1,5], Claire Mallard[2], Julien Carcaillet[1], Danny H. Natawidjaja[5], Didier Bourlès[3] and ASTER team[3]

[1*]ISTerre CNRS, Univ. Grenoble Alpes IRD, Grenoble, 38000, France.
[2]School of Geosciences, The University of Sydney, Sydney, 2006, NSW, Australia.
[3]CEREGE, Aix-Marseille Université CNRS-IRD-Collège de France-INRAE, Technopôle de l'Environnement Arbois-Méditerrannée, Aix-en-Provence, 13545, France.
[4]Research Center for Archaeometry, National Research and Innovation Agency (BRIN), Jakarta, Indonesia.
[5]Research Center for Geotechnology, National Research and Innovation Agency (BRIN), Bandung, Indonesia.

*Corresponding author(s). E-mail(s): laurent.husson@univ-grenoble-alpes.f;

Lithological facies	Sample	Depth (cm)	Dissolved quartz (g)	[^{26}Al] (10^5at.g^{-1})	[^{10}Be] (10^5at.g^{-1})	^{26}Al/^{10}Be
Tuff and silstone	SAN18-1	2.5	10.855	1.036 ± 0.178	0.296 ± 0.029	3.50 ± 0.69
	SAN18-2	12.5	7.700	1.141 ± 0.225	0.228 ± 0.029	5.02 ± 1.18
	SAN18-3	17.5	3.128	0.944 ± 0.313	0.210 ± 0.058	4.50 ± 1.94
	SAN18-4	60	3.817	1.245 ± 0.345	0.228 ± 0.047	5.46 ± 1.88
	SAN18-5	85	4.835	0.933 ± 0.225	0.261 ± 0.041	3.58 ± 1.03
	SAN18-6	32.5	1.223	0.907 ± 0.412	0.242 ± 0.123	3.75 ± 2.55
Tuffaceous sand	SAN18-10	190	0.869	1.850 ± 0.847	0.550 ± 0.226	3.37 ± 2.07
	SAN18-11b	240	3.345	0.646 ± 0.289	0.159 ± 0.050	4.05 ± 2.21
	SAN18-12	290	13.740	0.821 ± 0.123	0.277 ± 0.047	2.96 ± 0.67
	SAN18-12b	290	9.612	0.539 ± 0.237	0.224 ± 0.030	2.41 ± 1.11
Bedded sand and gravel	SAN18-13	350	12.479	0.857 ± 0.147	0.223 ± 0.034	3.85 ± 0.88
	SAN18-14	400	19.986	1.083 ± 0.137	0.289 ± 0.027	3.75 ± 0.59
	SAN18-15	450	20.004	0.792 ± 0.149	0.277 ± 0.026	2.85 ± 0.60
Conglomerate *Grenzbank*	SAN18-16	475	6.293	1.422 ± 0.399	0.261 ± 0.050	5.45 ± 1.85
	SAN18-17	500	15.904	0.734 ± 0.109	0.268 ± 0.029	2.74 ± 0.50
	SAN18-18	545	9.020	0.978 ± 0.222	0.239 ± 0.030	4.08 ± 1.06

Table 1 Analytical Data. ^{26}Al/^{10}Be analyses. For each sample, ∼150 μl of the an in-house phenakite 3.10^{-3} g.g^{-1} ^{9}Be carrier solution was added (S.I. Table 3). ^{27}Al natural concentrations were measured by ICP-OES, and for 8 samples a variable amount of an aluminum carrier solution (Chemlab 983.28 g.g^{-1}) was added. Concentrations were corrected for chemical blank (for samples SAN18-8 to SAN18-18, blank ratios were 1.93±0.34 x 10^{-15} and 1.93±1.3 x 10^{-15} for ^{10}Be/^{9}Be and ^{26}Al/^{27}Al ratios, respectively; for samples SAN18-1 to SAN18-6, SAN18-11b and SAN18-12b blank ratios were 1.58±0.35 x 10^{-15} and 1.06±1.05 x 10^{-15} for ^{10}Be/^{9}Be and ^{26}Al/^{27}Al ratios, respectively). Density is set to 2 g.cm^{-3}. Uncertainties (±1σ) include only analytical uncertainties. χ^2 at 95% confidence level, for ^{10}Be and ^{26}Al concentrations, and ^{26}Al/^{10}Be ratios are respectively 14, 14, and 9, while theoretical χ^2 (for n=16) is 25. The Mean Squared Weighted Deviation (MSWD) within the sample population are respectively 0.95, 0.93 and 0.64.

			End-member case: Total burial *without* post-production		Generic case: Burial *with* post-production				
Lithological facies	Sample	Depth (cm)	Burial age (Ma)	Denudation rate, source (m/Ma)	age (Ma)	Denudation rate, source (m/Ma)	Denudation rate, post-deposition	% [^{10}Be], post-burial	% [^{26}Al], post-burial
Tuff and silstone	SAN18-1	2.5	1.49 ± 0.30	46		54		22.8	47.4
	SAN18-2	12.5	0.76 ± 0.18	88		69		27.2	39.8
	SAN18-3	17.5	0.98 ± 0.42	85		78		28.3	46.2
	SAN18-4	60	0.58 ± 0.20	96		59		18.9	26.0
	SAN18-5	85	1.44 ± 0.42	53		53		14.2	29.9
	SAN18-6	32.5	1.35 ± 0.92	60		66		21.8	43.0
Tuffaceous sand	SAN18-10	190	1.54 ± 0.95	23		21		4.3	9.8
	SAN18-11b	240	1.20 ± 0.66	101		84		13.3	24.7
	SAN18-12	290	1.83 ± 0.42	41		49		7.1	17.6
	SAN18-12b	290	2.25 ± 1.04	41	1.78 ± 0.35	61	436	8.8	26.7
Bedded sand and gravel	SAN18-13	350	1.30 ± 0.30	68		55		8.2	15.3
	SAN18-14	400	1.35 ± 0.21	50		41		6.1	11.3
	SAN18-15	450	1.90 ± 0.40	39		46		6.1	14.5
Conglomerate Grenzbank	SAN18-16	475	0.59 ± 0.20	83		44		6.4	7.8
	SAN18-17	500	1.99 ± 0.37	39		50		6.2	14.8
	SAN18-18	545	1.18 ± 0.31	67		50		6.7	10.5

Table 2 Inversion model outputs, *without* (end-member case) and *with* post-burial production [1, 2]. The first case, considering no cosmogenic nuclides were accumulated in the samples while buried (infinite burial depth), yields minimum burial ages. The second, generic case, conversely considers cosmogenic nuclide production during post-burial exhumation, and yields an actual age. Uncertainties (reported as 1σ) obtained by propagating half-lives uncertainties. Parameters used for calculation: latitude: 7.47°; altitude: 99 m; pressure: 1001 mbar; mean density: 2 g.cm^{-3}; Stone scaling: 0.64; τ ^{10}Be: 1.387±0.0120 Ma [3, 4]; τ ^{26}Al: 0.705±0.024 Ma [5, 6]; P10 SLHL: 4.03±0.18 at.g^{-1}.a^{-1} [7, 8]; muon contributions and attenuation lengths are based on [2]. ^{26}Al/^{10}Be spallogenic production ratio: 6.61±0.52 [9].

Sample	Depth (cm)	Sample mass (g)	Dissolved pure quartz (g)	^{9}Be carrier (10^{19} at)	^{10}Be/^{9}Be (blank corrected)	ASTER total counts ^{10}Be	^{27}Al (10^{19} at.g^{-1})	^{27}Al carrier (10^{19} at)	^{26}Al/^{27}Al (blank corrected)	ASTER total counts ^{26}Al
SAN18-1	2.5	1224	10.86	3.022	1.064±0.104	202	0.575±0.011	0	1.697±0.327	34
SAN18-2	12.5	1181	7.70	3.048	0.575±0.073	135	0.569±0.011	0	1.899±0.409	34
SAN18-3	17.5	1910	3.13	3.021	0.217±0.060	59	1.313±0.026	2.204	0.614±0.261	11
SAN18-4	60	2084	3.82	3.077	0.283±0.058	109	1.066±0.021	1.813	1.063±0.341	15
SAN18-5	85	2128	4.83	3.043	0.414±0.065	131	0.837±0.017	1.255	1.010±0.289	19
SAN18-6	32.5	1737	1.22	3.058	0.097±0.049	54	3.361±0.067	3.265	0.165±0.162	5
SAN18-10	190	140	0.87	3.023	0.158±0.065	45	4.756±0.095	3.442	0.196±0.224	5
SAN18-11b	240	1197	3.35	3.044	0.175±0.055	61	1.660±0.033	0	0.283±0.203	5
SAN18-12	290	151	13.74	3.042	1.252±0.214	47	0.416±0.008	0	1.679±0.318	52
SAN18-12b	290	1421	9.61	3.051	0.912±0.137	110	0.450±0.009	0	1.611±0.348	7
SAN18-13	350	137	12.48	3.051	0.912±0.137	80	0.450±0.009	0	1.611±0.348	34
SAN18-14	400	194	19.99	3.057	1.887±0.173	170	0.385±0.008	0	2.517±0.373	68
SAN18-15	450	181	20.00	3.066	1.811±0.170	148	0.369±0.007	0	1.849±0.419	42
SAN18-16	475	292	6.29	3.050	0.539±0.103	80	0.644±0.013	1.111	2.015±0.634	26
SAN18-17	500	181	15.90	3.016	1.414±0.152	123	0.281±0.006	0	2.313±0.404	51
SAN18-18	545	140	9.02	3.026	0.714±0.090	123	0.448±0.009	0.842	1.991±0.515	27

Table 3 Complementary anaytical data. Uncertainties (± 1σ) include only analytical uncertainties. To each sample, ~150 μl of the LN2C in-house phenakite 3 10^{-3} g g^{-1} ^{9}Be carrier solution was added. ^{27}Al natural concentrations were measured by ICP-OES, and 8 of them were supplemented with a variable amount of an aluminum (Chemlab 983.28 g.g^{-1}) carrier solution sufficient to perform measurements. The concentration measurements were corrected for the chemical blank ratios for the first batch of (Samples SAN18-8 to 18) 1.93±0.34 10^{-15} and 1.93±1.3 10^{-15} for ^{10}Be/^{9}Be and ^{26}Al/^{27}Al ratios, respectively; and for the second batch (Samples SAN18-1 to 6 and SAN18-11b and SAN18-12b) 1.58±0.35 10^{-15} and 1.06±1.05 10^{-15} for ^{10}Be/^{9}Be and and ^{26}Al/^{27}Al ratios, respectively.

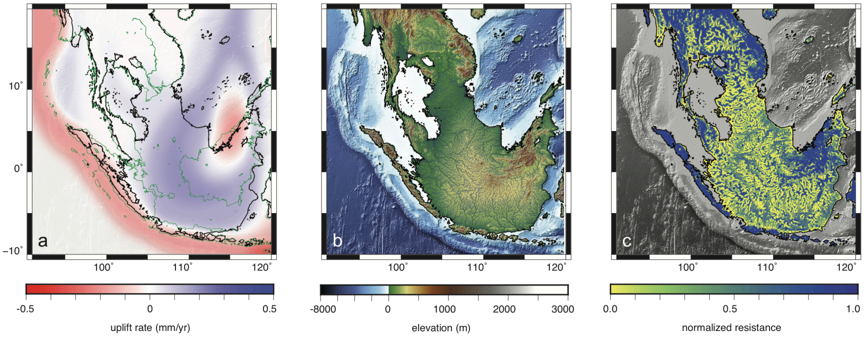

Fig. 1 Vertical land motion, physiography, and cost surfaces at 1.8 Ma. **a.** Uplift rates, inferred from geomorphological indicators, stratigraphic, and seismic data (adapted from [10, 11]). Green curves delineate present-day shorelines **b.** Reconstructed physiography from landscape evolution model [12]. River width scales with reconstructed water fluxes. **c.** normalized cost map based on distance to rivers and coastlines, river discharge, and topographic slopes, which quantifies the local resistance of the landscape to species displacement (cost values increase with resistance). All maps were made using GMT 5 (www.generic-mapping-tools.org) [13].

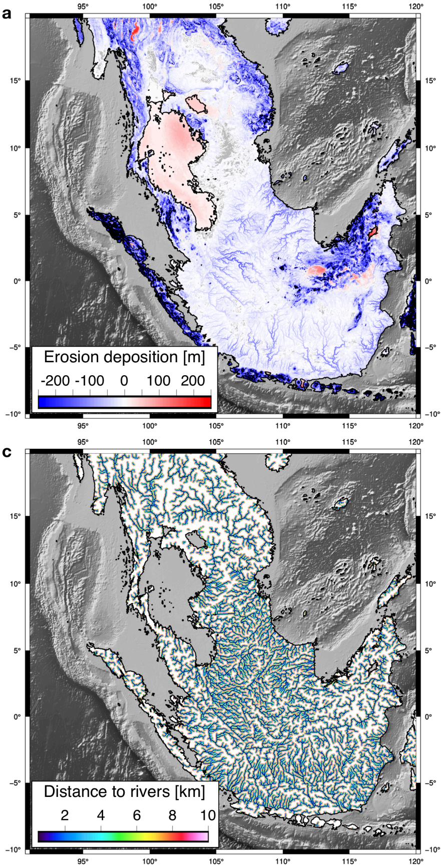

Fig. 2 Physiographic variables used to evaluate resistance to movement. **a.** Simulated cumulative erosion deposition after 10 kyr of landscape evolution induced by riverine and hillslope processes. **b.** Mean slopes for the continental region computed over the 1 km grid. **c.** Extracted distance to rivers (based on the distribution presented in Fig. 1b) used to estimate riparian areas. **d.** Position of the largest rivers defined with a flow rate above 5.5×10^3 m^3/s and used to impede movement across the region. All maps were using GMT 5 (www.generic-mapping-tools.org) [13].

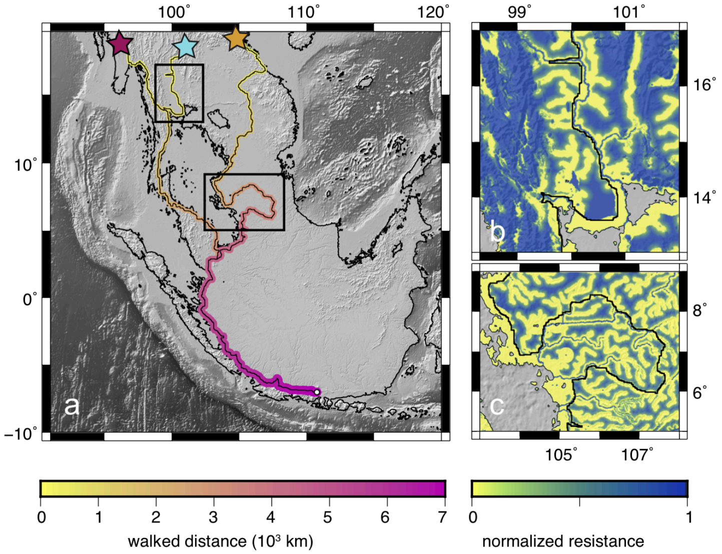

Fig. 3 Least-cost path routes to Sangiran. **a.** Computed shortest walked distance assuming 3 different north entry points (Myanmar, Thailand, Vietnam). **b., c.** Zoomed-in subregions, where the role of the cost surface on calculated paths is highlighted. Black lines correspond to the least-cost paths for the central and eastern routes respectively. All maps were using GMT 5 (www.generic-mapping-tools.org) [13].

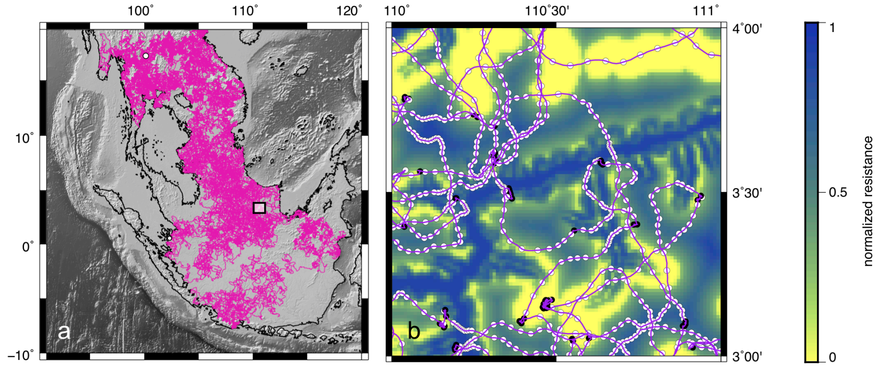

Fig. 4 Markovian walk of *H. erectus* across Sundaland, from the mechanistic spatially-explicit simulation SiMRiv [14]. **a.** Sample realization (central northern entry point) for 5 millions steps. **b.** 1° box (white shaded area in **a**) showing positions every 25 steps and illustrating *H. erectus* dynamics with two-state movements (random walk in purple, and correlated random walk in white) and dependency on landscape heterogeneity. All maps were using GMT 5 (www.generic-mapping-tools.org) [13].

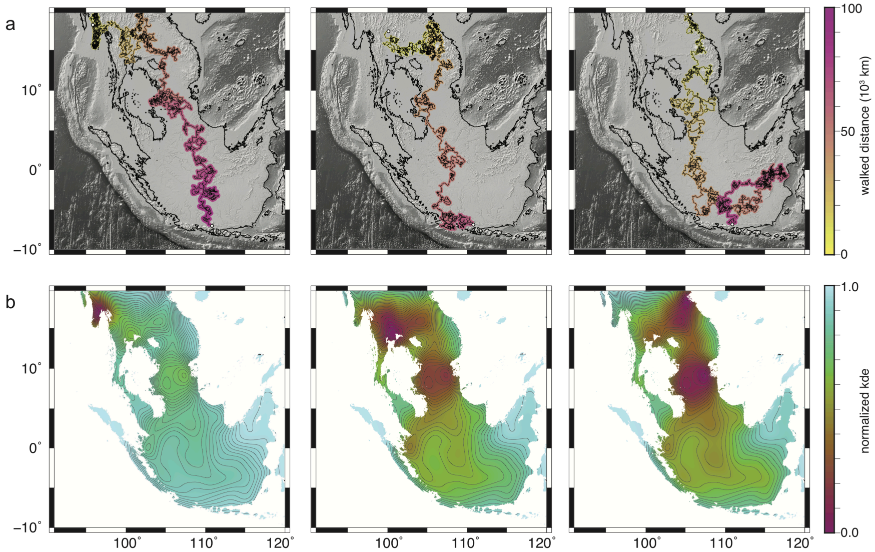

Fig. 5 Successful Markovian walks to Sangiran. **a.** Shortest walked distance (least cost path) realizations for each set of 1000 realizations (for the 3 entry points). **b.** Normalized kernel density estimates (kde) from predicted positions obtained from the mechanistic model and inversely weighted based on the normalized cost surface (Fig. 1c). All maps were using GMT 5 (www.generic-mapping-tools.org) [13].

References

[1] Pappu, S. *et al.* Early Pleistocene Presence of Acheulian Hominins in South India. *Science* **331** (6024), 1596–1599 (2011) .

[2] Braucher, R., Merchel, S., Borgomano, J. & Bourlès, D. Production of cosmogenic radionuclides at great depth: A multi element approach. *Earth and Planetary Science Letters* **309** (1), 1–9 (2011) .

[3] Chmeleff, J., von Blanckenburg, F., Kossert, K. & Jakob, D. Determination of the 10Be half-life by multicollector ICP-MS and liquid scintillation counting. *Nuclear instruments & methods in physics research. Section B, Beam interactions with materials and atoms* **268** (2), 192–199 (2010) .

[4] Korschinek, G. *et al.* A new value for the half-life of 10Be by Heavy-Ion Elastic Recoil Detection and liquid scintillation counting. *Nuclear instruments & methods in physics research. Section B, Beam interactions with materials and atoms* **268** (2), 187–191 (2010) .

[5] Norris, T. L., Gancarz, A. J., Rokop, D. J. & Thomas, K. W. Half-life of 26Al. *Journal of Geophysical Research: Solid Earth* **88** (S01), B331–B333 (1983) .

[6] Nishiizumi, K. Preparation of 26Al AMS standards. *Nucl. Inst. and Meth. in Phys. Res.* **223-224**, 388–392 (2004) .

[7] Molliex, S. *et al.* Quaternary evolution of a large alluvial fan in a periglacial setting (Crau Plain, SE France) constrained by terrestrial cosmogenic nuclide (10Be). *Geomorphology* **195**, 45–52 (2013) .

[8] Borchers, B. *et al.* Geological calibration of spallation production rates in the CRONUS-Earth project. *Quaternary Geochronology* **31**, 188–198 (2016) .

[9] Rixhon, G. *et al.* Quaternary river incision in NE Ardennes (Belgium)–Insights from 10Be/ 26Al dating of river terraces. *Quaternary Geochronology* **6** (2), 273–284 (2011) .

[10] Salles, T. *et al.* Quaternary landscape dynamics boosted species dispersal across Southeast Asia. *Communications Earth & Environment* **2** (1), 1–12 (2021) .

[11] Husson, L. *et al.* Slow geodynamics and fast morphotectonics in the far East Tethys. *Geochemistry, Geophysics, Geosystems* **23** (1), n/a (2022) .

[12] Salles, T., Mallard, C. & Zahirovic, S. gospl: Global Scalable Paleo Landscape Evolution. *Journal of Open Source Software* **5** (56), 2804 (2020).

https://doi.org/10.21105/joss.02804 .

[13] Wessel, P., Smith, W. H. F., Scharroo, R., Luis, J. & Wobbe, F. Generic mapping tools: Improved version released. *Eos, Transactions American Geophysical Union* **94** (45), 409–410 (2013). URL https://agupubs.onlinelibrary.wiley.com/doi/abs/10.1002/2013EO450001. https://doi.org/https://doi.org/10.1002/2013EO450001, https://arxiv.org/abs/https://agupubs.onlinelibrary.wiley.com/doi/pdf/10.1002/2013EO450001 .

[14] Quaglietta, L. & Porto, M. SiMRiv: An R package for mechanistic simulation of individual, spatially-explicit multistate movements in rivers, heterogeneous and homogeneous spaces incorporating landscape bias. *Movement Ecology* (2019). https://doi.org/10.1186/s40462-019-0154-8 .
